# Intercalary Reconstruction after Wide Resection of Malignant Bone Tumors of the Lower Extremity Using a Composite Graft with a Devitalized Autograft and a Vascularized Fibula

**DOI:** 10.1155/2015/861575

**Published:** 2015-02-16

**Authors:** Koichi Ogura, Shimpei Miyamoto, Minoru Sakuraba, Tomohiro Fujiwara, Hirokazu Chuman, Akira Kawai

**Affiliations:** ^1^Department of Musculoskeletal Oncology, National Cancer Center Hospital, 5-1-1 Tsukiji, Chuo-ku, Tokyo 104-0045, Japan; ^2^Department of Plastic and Reconstruction Surgery, National Cancer Center Hospital, 5-1-1 Tsukiji, Chuo-ku, Tokyo 104-0045, Japan

## Abstract

*Introduction*. Although several intercalary reconstructions after resection of a lower extremity malignant bone tumor are reported, there are no optimal methods which can provide a long-term reconstruction with fewest complications. We present the outcome of reconstruction using a devitalized autograft and a vascularized fibula graft composite.* Materials and Methods.* We conducted a retrospective review of 11 patients (7 males, 4 females; median age 27 years) undergoing reconstruction using a devitalized autograft (pasteurization (*n* = 6), deep freezing (*n* = 5)) and a vascularized fibula graft composite for lower extremity malignant bone tumors (femur (*n* = 10), tibia (*n* = 1)).* Results*. The mean period required for callus formation and bone union was 4.4 months and 9.9 months, respectively. Four postoperative complications occurred in 3 patients: 2 infections (1 pasteurized autograft, 1 frozen autograft) and 1 fracture and 1 implant failure (both in pasteurized autografts). Graft removal was required in 2 patients with infections. The mean MSTS score was 81% at last follow-up.* Conclusions.* Although some complications were noted in early cases involving a pasteurized autograft, our novel method involving a combination of a frozen autograft with a vascularized fibula graft and rigid fixation with a locking plate may offer better outcomes than previously reported allografts or devitalized autografts.

## 1. Introduction

Limb salvage surgery has replaced amputation for malignant musculoskeletal tumors. Most bone sarcomas occur in the metaphyseal portion of the bone and typical resection involves the whole proximal or distal part of the bone. Therefore, in most cases, the resected segment of bone is replaced by a prosthesis, which provides satisfactory results quickly after surgery. However, when the tumor involves the diaphyseal portion of the bone, an intercalary reconstruction method is required, and this has not been standardized. Intercalary allografts, which is the most widely accepted reconstruction method, are associated with high incidences of nonunion (12–57%), fracture (17–30%), and infection (10–15%). [1–6]. In addition, single use of devitalized autograft such as frozen autograft or pasteurized autograft was also associated with nonunion (20% and 7%, resp.) [7, 8]. Although a segmental prosthesis can provide immediate stability and good short-term postoperative function, it is associated with long-term problems including implant wear, breakage, and loosening with the 10-year implant survival of 63% [9]. Therefore there is an urgent need to develop an optimal reconstruction method for this type of condition.

Vascularized fibula grafts have been reported to yield favorable outcomes in terms of bone union in cases of trauma, infection, or musculoskeletal tumors [10–15]. Recently, several investigators have reported an intercalary reconstruction technique using a composite graft comprising a free vascularized fibula graft with an allograft, or an extracorporeally irradiated or pasteurized autograft, in order to overcome shortcomings such as nonunion, infection, and fracture resulting from deterioration of mechanical strength [10, 16–18].

The aim of the present study was to analyze the clinical and functional outcomes of intercalary reconstruction using a composite graft comprising a devitalized autograft and a vascularized fibula graft after wide resection of malignant bone tumor of the lower extremity and compare the results with those reported previously for other reconstruction methods. In addition, we investigated the applicability of a novel surgical technique using a frozen autograft and a vascularized fibula graft composite and compared it with pasteurized autograft group.

## 2. Patients and Methods

We conducted a retrospective review of 11 consecutive patients who underwent intercalary bone defect reconstruction using a devitalized autograft combined with a vascularized fibula graft composite between 2007 and 2011. Their clinical data, treatment modalities, and outcome were reviewed retrospectively with reference to the medical records. The mean follow-up period was 68 months (range, 25–131 months).

The following data were examined: demographic data (patient age at operation, gender, tumor site, and histologic diagnosis), surgical details (length of bone defect, methods of devitalization, operation time, total blood loss, and reconstruction details), adjuvant therapy (chemotherapy and radiotherapy), postoperative complications (nonunion, fracture, implant failure, or infection), the time required for bone union, the findings of bone scintigraphy, oncologic outcomes, and functional outcomes.

**Figure 1:**
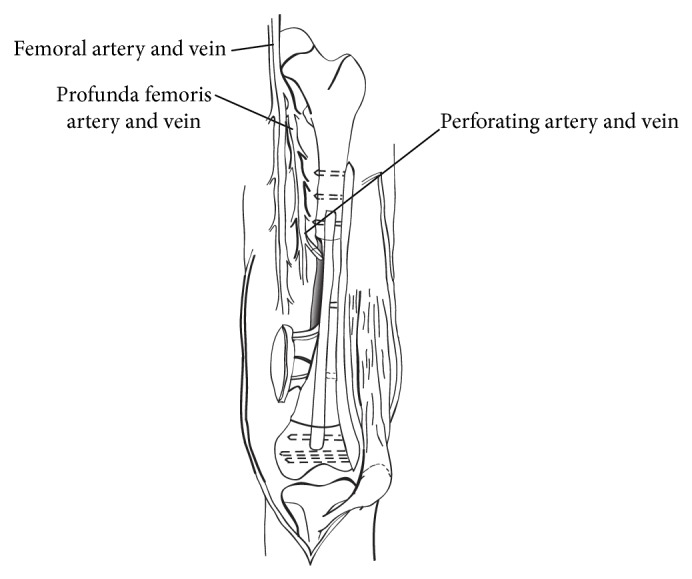
Illustration showing reconstruction of a femur diaphysis using a composite graft with a devitalized autograft and a vascularized fibula graft.

Wide resection of the tumor was performed in all cases, and the bone defect was reconstructed with a devitalized autograft and a vascularized fibula graft placed into the medullary canal of the autograft. The composite graft was rigidly fixed with a plate and screws (Figure 1). The pasteurized or frozen autografts were prepared as described previously [7, 19, 20]. Bone union was defined as the presence of fusion between the host bone and the devitalized autograft at both ends and full weight-bearing without pain. Bone scintigraphy was performed soon after surgery (within 2 months) and late after surgery (more than 6 months) in selected cases. Functional outcome of the reconstructed limb was assessed using the Musculoskeletal Tumor Society (MSTS) scoring system [21], which included pain, function, emotional acceptance, use of any external support, walking ability, and gait alteration.

## 3. Results

Patient demographics and treatment data are summarized in Tables 1 and 2. There were 7 males and 4 females with a mean age of 29 years (range, 11–63 years). No patients had preoperative comorbidities that might have influenced the bone healing time. The histological diagnoses were osteosarcoma (*n* = 6), bone involving soft tissue sarcoma (*n* = 3), Ewing's sarcoma (*n* = 1), and chondrosarcoma (*n* = 1). The locations of the resected bone were the femur (*n* = 10) and tibia (*n* = 1). Adjuvant chemotherapy and radiotherapy were performed in 8 and 1 patients, respectively. The mean length of the bone defect was 19 cm (range, 10–22 cm). The methods of devitalization included pasteurization (*n* = 6) and deep freezing (*n* = 5). Free bone grafting from iliac crest at the junction was performed in 8 patients. The fixation method included a plate in 5 patients, a locking plate in 5 patients, and only screws in 1 patient. The total operation time ranged from 430 to 910 minutes (mean, 698 minutes) and mean blood loss was 1,086 mL (range, 354–2,162 mL). Although the mean operation time was shorter and mean blood loss was less in the frozen autograft group (645 minutes, 841 mL) than in the pasteurized autograft group (710 minutes, 1,291 mL), the differences were not statistically significant (*P* = 0.511 and 0.319, resp.).

Treatment results are summarized in Table 3. Bone union was achieved in 10 patients (91%). In case 5, deep infection occurred and resulted in graft removal to cure the infection at 2 months after surgery. Therefore, bone union was not evaluable. The mean period required for callus formation and bone union was 4.4 months (range, 3–11 months) and 9.9 months (range, 4–14 months), respectively. Time required for bone union was significantly shorter in the frozen autograft group (7.0 months) than in the pasteurized autograft group (11.2 months) (Student's *t*-test, *P* = 0.042). Although no statistical significance was noted, the mean period until bone union in patients who underwent free bone grafting was shorter than in those who did not (8.9 months versus 11.0 months; Student's *t*-test, *P* = 0.320). Hypertrophy of the vascularized fibula and subsequent bone integration into the devitalized autograft was seen in only 2 patients at 21 months (Patient 4) and 39 months (Patient 7) after surgery, respectively. Bone scintigraphy in the early phase was performed in 7 patients and all of them showed increased uptake in the fibula graft and no uptake in the devitalized autograft. Among these patients, 3 underwent bone scintigraphy in the late phase; 2 patients showed increased uptake in both the fibula graft and the devitalized autograft, and 1 patient showed increased uptake in the fibula graft and no uptake in the devitalized autograft.

Four postoperative complications occurred in 3 patients: 2 infections (1 pasteurized autograft, 1 frozen autograft) and 1 fracture at the proximal host-graft junction and 1 implant failure (screw breakage) (both in pasteurized autografts). No significant difference in the postoperative complication rate was seen between the pasteurized and frozen autograft groups (Fisher's exact test, *P* = 0.621). Graft removal was required in 2 patients with infections. Two patients suffered from local recurrence out of the pasteurized autograft, which necessitated amputation. Oncological outcomes for the patients overall were CDF in 5, NED in 1, AWD in 3, and DOD in 2 patients at the time of last follow-up. The mean MSTS score was 81% (range, 43–100%). The mean MSTS score was relatively higher in the frozen autograft group (85%) than in the pasteurized autograft group (70%), but the difference was not statistically significant (Student's *t*-test, *P* = 0.211).

**Figure 2:**
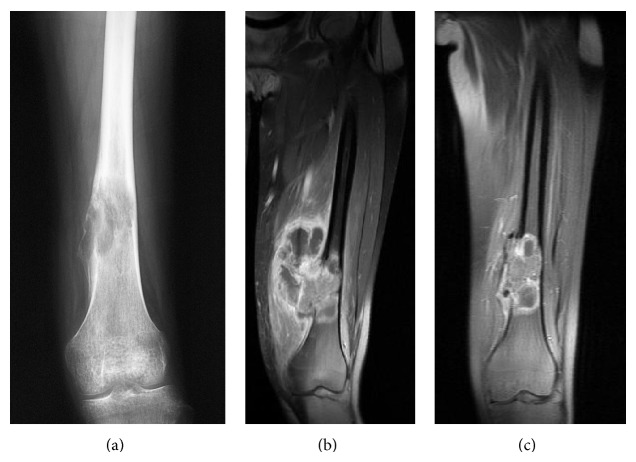
A plain radiograph of the left distal femur demonstrates an osteolytic lesion with destruction of the cortex and intratumoral ossification (a). Coronal MR images demonstrate a large extraosseous mass with destruction of the cortex (b). Reduction in size of the tumor was noted after preoperative chemotherapy (c).

**Figure 3:**
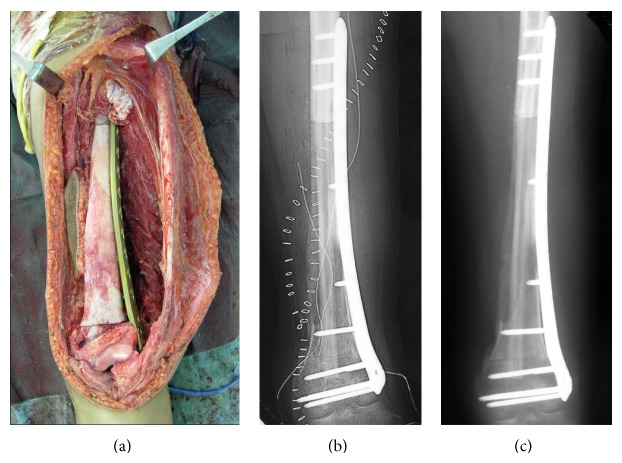
The composite graft was rigidly fixed to the host bone with a locking plate and screws ((a) operative photograph, (b) postoperative plain radiograph). A plain radiograph 9 months after surgery. Bone union was achieved (c).

**Figure 4:**
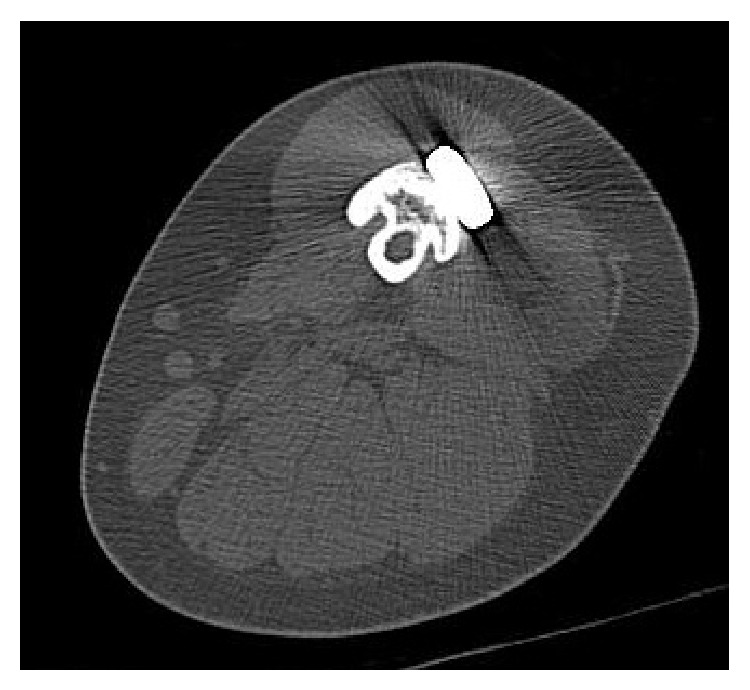
A CT scan at 39 months after surgery. Bridging bone formation from the hypertrophic inlaid fibula to the frozen autograft is evident.

*Representative Case (Patient 7)*. The patient was a 16-year-old female with osteosarcoma of the left distal femur (Figure 2(a)). She underwent neoadjuvant chemotherapy with adriamycin, cisplatin, and ifosfamide, and reduction in the size of the tumor was noted (Figures 2(b) and 2(c)). Intercalary wide resection was performed and the length of the bone defect was 20 cm. This was reconstructed using a composite graft comprising a frozen autograft and a vascularized fibula placed into the medullary canal of the frozen autograft. Microvascular anastomoses were performed and the composite graft was finally fixed to the host bone with a locking plate and screws (Figures 3(a) and 3(b)). Postoperatively, adjuvant chemotherapy with the same agents was performed. There were no postoperative complications or local recurrence. At 9 months after surgery, bone union at the host-devitalized autograft junction and host-fibula graft junction was achieved (Figure 3(c)). Hypertrophy of the inlaid fibula and subsequent integration with the frozen autograft became evident on CT at 39 months after surgery (Figure 4). The patient was able to walk without a walking aid and enjoy mild sports activity at 75 months after surgery. The MSTS score at the time of last follow-up was 100%.

## 4. Discussion

Reconstruction options following intercalary resection of lower extremity malignancy have included the use of massive allografts [22–24], autoclaved autografts [25], pasteurized autografts [8], irradiated autografts [26], and segmental prostheses [9, 27]. Each procedure has its own shortcomings and no standardized method has been established. The complication rate associated with intercalary allograft reconstruction has been considerably high. The complications of intercalary allografts have included nonunion (12–57%), fracture (17–30%), and infection (10–15%) [1–6, 22–24, 28]. One of the main disadvantages of devitalized autografts such as autoclaved, pasteurized, or irradiated autografts is that it takes a long time for them to be revascularized and incorporated into the surrounding bone. In addition, several investigators have reported high rates of infections, fractures, nonunions, and bone resorption associated with the procedure [8, 25, 26, 28]. For example, reconstruction using a pasteurized autograft was associated with high rate (52%) of complications including primary nonunion (20%), infection (20%), fracture (12%), and massive bone resorption (8%) [8]. Reconstruction using an autograft frozen with liquid nitrogen was reported to be a simple and effective method of biological reconstruction [7]. However, it still has some problems similar to those of allografts or other types of devitalized autografts, including infection (11%), fracture (7%), or nonunion (7%) [7], and there is an urgent need to develop an optimal reconstruction to decrease these problems.

Recently, a composite graft technique that combines an allograft or a devitalized autograft with a vascularized fibula graft for reconstruction of large bony defects has been developed [10, 16–18, 29, 30]. The rationale for use of a composite graft is that it combines the advantages of immediate mechanical endurance of a massive allograft or a devitalized autograft with the long-standing biological properties of a vascularized fibula graft. The allograft or devitalized autograft provides a massive bone stock and early stabilization, whereas the vascularized fibular graft facilitates host-graft union. Chang and Weber [18] reported 6 primary intercalary reconstructions with composite use of a vascularized fibula graft and an allograft. All of the 6 studied patients (100%) achieved successful bone union, and the mean time until bone union was 6 months (range, 3–8 months). Sugiura et al. [10] reported 15 intercalary reconstructions with composite use of a vascularized fibula graft and a pasteurized autograft. Bone union was achieved in 13 cases (87%) and the mean period until bone union was 13.5 months (range, 7–25 months). Postoperative complications included fractures in 2 patients and infection in another 2. There were no local recurrences. Krieg et al. [17] reported 13 intercalary reconstructions with composite use of a vascularized fibula graft and an extracorporeal irradiated autograft. Nonunion was found in 16% of host-donor junctions and they required additional surgical procedures. The median time until primary bone union was 7.5 months at metaphyseal junctions and 11.1 months at diaphyseal junctions. Postoperative complications included 1 fracture. There were no cases of infection. Although direct comparison is difficult due to the lack of statistical power and the heterogeneity of the population, the bone union rate in the present study (91%) was comparable to these reports (84–100%) and postoperative complication rate (27%) seemed relatively high compared with these reports (0–27%) [10, 17, 18].

In addition to reconstruction with a previously reported combination of a vascularized fibula graft and a pasteurized autograft, we performed a novel reconstruction method combining a vascularized fibula graft with a frozen autograft for large segmental bone defects. Although there were no significant differences, perhaps as a result of limited statistical power, the frozen autograft group tended to have a shorter operation time, less blood loss, a lower postoperative complication rate, and better functional outcome. Out of 3 postoperative complications in 2 patients in the pasteurized autograft group, 1 fracture and 1 implant failure were related to insufficient internal fixation, and hence they may have been avoided by rigid fixation using a locking plate, as used in recent cases involving frozen autografts. The better functional outcome in the frozen autograft group may have been associated with earlier bone union, resulting in early mobilization of the reconstructed limb postoperatively. Another major point with regard to the use of a frozen autograft in composite graft reconstruction is its ability to be used anywhere and the simplicity of the procedure.

However, reconstruction using a composite graft has several disadvantages. First, it is impossible to perform histological analysis of the whole specimen for determining the effects of chemotherapy and the adequacy of the surgical margins. Second, it takes a longer operation time and requires specialist plastic surgeons. Third, the majority of the cases in the present study consisted of the reconstruction of the femur, which is considered to be less challenging than that of the tibia. Further investigation is required to show the applicability to expand this technique to patients with reconstruction of the tibia. Finally, some donor site complications which require surgical intervention such as claw toe deformity or peroneal nerve palsy may occur after the harvesting of vascularized fibula graft although there were no cases of such complications requiring surgical intervention in our series.

## 5. Conclusions

In conclusion, although some complications were noted especially in early cases involving a pasteurized autograft, our results suggest that a devitalized autograft combined with a vascularized fibula graft is a promising biological alternative for intercalary reconstruction after wide resection of malignant bone tumors of the lower extremity, especially in younger patients for whom longer life expectancy and increased physical activity make fractures and infections more likely. In addition, our novel method involving a combination of a frozen autograft with a vascularized fibula graft and rigid fixation with a locking plate may offer better outcomes than previously reported allografts or devitalized autografts, although longer follow-up of a greater number of patients will be required to confirm this.

## Figures and Tables

**Table 1:** Patient demographics and adjuvant therapy data.

Number	Age	Gender	Histologic diagnosis	Tumor site	Tumor size (cm)	Chemotherapy	Radiotherapy
1	20	M	Osteosarcoma	Femur	16	Preoperative/postoperative	None
2	11	F	Osteosarcoma	Femur	16	Preoperative/postoperative	None
3	49	M	Bone involving recurrent myxoid liposarcoma	Femur	14	Preoperative/postoperative	Preoperative
4	27	M	Fibrosarcoma (grade 2)	Femur	13	None	None
5	37	M	Ewing's sarcoma	Femur	13	Preoperative/postoperative	None
6	29	F	Parosteal osteosarcoma	Femur	6	None	None
7	16	F	Osteosarcoma	Femur	14	Preoperative/postoperative	None
8	36	M	Osteosarcoma	Tibia	10	Preoperative/postoperative	None
9	11	F	Osteosarcoma	Femur	16	Preoperative/postoperative	None
10	63	M	Bone involving myxofibrosarcoma	Femur	12	Preoperative	None
11	19	M	Chondrosarcoma (grade 2)	Femur	16	None	None

**Table 2:** Surgical details of the patients.

Number	Length of bone defect (cm)	Method of devitalization	Free bone graft at the junction	Fixation	Operation time (min)	Blood loss (mL)
1	22	Pasteurization	Yes	Plate	630	1615
2	22	Pasteurization	Yes	Plate	540	574
3	20	Pasteurization	Yes	Plate	670	713
4	18	Pasteurization	No	Plate	910	1900
5	20	Pasteurization	No	Plate	660	823
6	10	Pasteurization	No	Screw	848	2121
7	20	Deep freezing	Yes	Locking plate	430	634
8	16	Deep freezing	Yes	Locking plate	854	604
9	22	Deep freezing	Yes	Locking plate	684	354
10	18	Deep freezing	Yes	Locking plate	750	450
11	22	Deep freezing	Yes	Locking plate	506	2162

**Table 3:** Treatment, oncologic, and functional outcomes.

Number	Time to callus formation (months)	Time to bone union (months)	Postoperative complications	Additional surgery for complications	Graft removal or amputation	Local recurrence	Metastasis	Oncologic outcome	Follow-up period (months)
1	3	7	Graft displacement resulting from screw breakage (11 months)	Reduction and refixation	Amputation due to recurrence	Yes	Lung, bone	DOD	44
2	4	12	None		No	No	No	CDF	131
3	3	13	None		Amputation due to recurrence	Yes	No	NED	129
4	11	14	None		No	No	Lung	DOD	39
5	6	12	Fracture (21 months) Infection (32 months)	Reduction and refixation Graft removal	Removal due to deep infection	No	No	CDF	104
6	4	10	None		No	No	No	CDF	81
7	3	9	None		No	No	No	CDF	75
8	NA	NA	Infection (2 months)	Graft removal and external fixation	Removal due to deep infection	No	Lung	AWD	56
9	4	10	None		No	No	Lung	AWD	37
10	3	8	None		No	No	Lung	AWD	31
11	3	4	None		No	No	No	CDF	25
